# Rural admission quota in Germany: students’ attitudes, challenges and the need for support programmes

**DOI:** 10.1186/s12909-026-08776-w

**Published:** 2026-02-11

**Authors:** Maike Krauthausen, Lisa Wingender, Veronika Deyerl, Pamina E. Hagen, Tobias Leutritz, Anne Simmenroth

**Affiliations:** 1 Department of General Practice, University Hospital Würzburg, Würzburg, Germany; 2 Institute for Medical Teaching and Medical Education Research, University Hospital Würzburg, Würzburg, Germany

**Keywords:** Rural doctor quota; study admission, Medical studies, Rural primary care, General practice/ family medicine

## Abstract

**Background:**

The shortage of general practitioners, especially in rural and underserved areas, is a pressing issue worldwide and also in Germany. To address this, several German federal states have introduced the Rural Doctor Quota (RDQ), which allocates study places in advance in exchange for a commitment to complete specialist training and work as a family doctor in a rural region. Despite its implementation, little is known about students’ attitudes toward the RDQ or the potential burdens associated with quota admission. This study provides the first cross-location description of RDQ students in Bavaria across all five medical faculties, focusing on their attitudes and on the assessment of potential burdens associated with this quota.

**Methods:**

A cross-sectional online survey was conducted among RDQ students and a control group of other students in their first to fourth year at all five Bavarian medical faculties. Participants completed Likert-scale items on attitudes towards rural practice and stress related to contractual obligations and indirect effects in association with the quota, complemented by free-text responses. Quantitative data were analysed descriptively, while free text responses were processed via structured content analysis, following Kuckartz, to identify core themes.

**Results:**

Of 359 respondents, 158 were students admitted through the Bavarian rural doctor quota (RDQ), and 201 were Non-RDQ students. RDQ students expressed positive attitudes towards rural general practice. Stress due to contract obligations was highest regarding uncertainty about future working regions and time commitment, while specialty and rural placement restrictions were less burdensome. Social stigma was stated to be low, though concerns about commitment or socio-economic advantages were expressed by Non-RDQ students. RDQ students reported higher financial strains and more study-work conflicts.

**Conclusion:**

RDQ students face particular challenges and need targeted support in the form of structured and multi-layered university programmes, next to financial support options. Rural placement, an in-depth curriculum and social network support can further promote students’ positive attitudes towards rural general practice and their commitment to the programme.

**Supplementary Information:**

The online version contains supplementary material available at 10.1186/s12909-026-08776-w.

## Introduction

The shortage of general practitioners (GPs), especially in rural and underserved areas, has become a pressing global issue, affecting both developed and developing countries alike. As the ageing population continues to grow, the demand for primary care is steadily rising [1, 2]. In Germany, however, one in three currently practicing GPs is already 60 years or older, further exacerbating concerns about future healthcare provision in rural regions [3]. At the same time, the number of newly trained physicians is insufficient to meet the increasing need. Many young doctors in Germany pursue careers outside the healthcare sector or choose medical specialties other than general practice (GP) [4–6].Compared to previous generations, over 60% of today’s GP trainees in Germany are women [6]. When choosing general practice as a career path, many prefer part-time work to achieve a better work-life balance and to combine professional and family commitments. At the same time, younger GPs of both genders increasingly value flexible working arrangements and are more likely to seek employed positions within existing practices rather than assuming entrepreneurial responsibilities [6]. Overall, forecasts suggest that Germany will face a shortage of more than 10,500 primary care physicians by 2030, particularly in rural regions [7]. The measures taken so far by politicians and local authorities include different areas of action, e.g. financial incentives, making employment conditions more flexible, strengthening the field of general practice in training and further education, increasing student numbers and developing and utilising the competence profiles of other health professions, as well as other areas of action [8–10].

One approach involves the targeted selection of students entering medical school who receive a placement in medical studies, the so-called ‘rural doctor quota’ (RDQ) in Germany (‘Landarztquote’- LAQ) [11]. This implies that up to 10% of study places go to applicants who express a particular interest in working as a general practitioner/family doctor (GP/FD) in rural areas. Admission to the programme comes with the obligation to complete the specialty training and then to work as a GP in an underserved region for at least ten years. This obligation is governed by a contract, and failure to comply with it will result in a penalty of up to €250,000. North Rhine-Westphalia was the first federal state to introduce the rural doctor quota in the 2019/20 winter semester. Most of the 16 federal states in Germany have since introduced a similarly structured quota [12]. To date, there is no standardised selection process at the federal level and no uniform regulations governing how the individual federal states in Germany implement the quota, whether they implement it at all and what selection criteria they use to choose applicants, and to what extent. In Bavaria, the selection process is identical at all medical faculties and consists of two stages: a points-based evaluation of test results, medical-related vocational training, and relevant voluntary work, followed by a structured interview to assess motivation and suitability, while high school grades are irrelevant [13, 14]. This applies, for example, to university entrance qualifications, which - in contrast to the regular university admission procedure - are not taken into account at all in many federal states when allocating places to RDQ students. With the exception of the federal state of Hesse, it is left to the universities to decide whether and to what extent they offer a supporting programme for RDQ students. Some universities provide offers for students interested in general practice [15, 16], but use individual approaches and are supported to varying degrees by the federal states.

The empirical study of the rural doctor quota in Germany is still in its early stages. In 2025, a first study by Geier et al. [17] focused on RDQ students’ characteristics in the federal state of Saxony. The authors found that students admitted via the rural doctor quota were more likely to come from rural backgrounds, have prior healthcare experience, and already show a stronger interest in general practice and running their own practice compared to Non-RDQ students. However, not all RDQ students were firmly committed to rural GP yet, so long-term outcomes remain uncertain.

In this study, we aim to provide the first cross-location description of the study situation of students in the rural doctor quota programme in Bavaria at all five medical faculties. The main focus is on students’ attitudes towards the rural doctor quota and on the description of potential burdens associated with this quota.

## Methods and material

### Study design and reporting guideline

This was an exploratory study involving an anonymous quantitative survey with the option of exploring individual topics in greater depth via open-ended questions with free-text responses. The following description of the survey part is in line with the ‘Checklist for reporting results of internet E-surveys’ (CHERRIES) [18].

### Design, approval, and informed consent process

The survey comprised items concerning students’ demography, study permission, influences on choosing medical studies, and aspects of the study process. The research was conducted in full compliance with the principles outlined in the Declaration of Helsinki; participants provided informed consent by reviewing the study information and proceeding to the survey. Participation in this study was voluntary and could be revoked at any time.

### Development and pre-testing

The survey was conducted online using the web-based survey software EvaSys^®^ (version 8.0, Electric Paper Evaluationssysteme GmbH, Lüneburg, Germany), which is hosted by the university. The questionnaire was developed on the basis of extensive literature research and piloted by research assistants at the Department of General Practice and non-quota students in Wuerzburg. A pre-test was carried out to eliminate any potential errors in the technical implementation. The questionnaire was specifically developed for this study and has not been published elsewhere (see supplement for an English translation).

### Recruitment process and selection of study subjects

All Bavarian university locations were involved: Munich, Erlangen, Augsburg, Regensburg and Wuerzburg. The sample consisted of students from the rural doctor quota (RDQ students) as well as a control group consisting of students who obtained their place at university through the regular or another selection process (Non-RDQ students). All students were in their first to fourth study year at most, as this was the maximum number that could be reached since the quota was introduced in Bavaria in 2020.

### Survey administration

Invitations to participate were distributed in multiple ways. The RDQ students were contacted in collaboration with the Bavarian State Office for Health and Food Safety (LGL), which is responsible for the selection process and the administration of the RDQ group. LGL forwarded an invitation letter containing a link to the online questionnaire to all RDQ students through its official email distribution list. After four weeks, a reminder email was sent by the LGL to encourage participation. Students in the control group were invited through the Departments of General Practice at the participating universities and through the respective local student councils. The survey was conducted over an eight-week period during the beginning of the 2024 summer semester (23th April – 17th June 2024).

### Response rates and preventing multiple entries from the same individual

According to the LGL, 434 students were enrolled under the rural doctor quota in Bavaria at the time of the survey. The exact number of students contacted via the student councils and medical faculties could not be determined. Multiple survey participation could not be completely ruled out, but checking the raw data did not reveal any evidence of systematic multiple participation.

### Study variables

As part of the extensive survey on the study situation of RDQ students, we focused on three areas: demographic and study admission data, attitudes toward the rural doctor quota and related aspects that are perceived as potentially stressful, such as quota obligations or financing of the studies. Most items were designed as 5-point-Likert scales. At the end of each section, participants had the opportunity to make further comments on the topic and freely add their own points. In total, a maximum of 215 items was presented; filters were used to vary the actual number of questions asked depending on individual characteristics. For open questions, the response was not mandatory and could be left empty. Reviewing the entries before final submission was possible. An excerpt of the items relevant to the study from the survey is presented in Appendix A.

### Data analysis

In a first step, quantitative survey data were calculated descriptively using IBM SPSS version 26 [19]. For mean comparisons between groups, two-sided independent samples t-tests were performed with a significance level of 0.05. Frequencies were analysed using cross-tabulations, and two-sample tests for equality of proportions were applied to compare the proportion of *sources of study funding.* Free text responses were evaluated by summary content analysis according to Kuckartz, a structured method for reducing and interpreting qualitative text data [20]. Texts were thoroughly read and based on this, a set of main codes and subcodes was developed (see Appendix B). Only one author of this study was involved in the evaluation of the free text responses (VD) During the coding process, the set of main codes and subcodes was further refined and complemented. For coding, we used the programme MAXQDA (VERBI GmbH, Berlin, Germany, Version 2024).

### Data management and data protection

In compliance with the European General Data Protection Regulation (GDPR), written informed consent was obtained. All data were collected and processed anonymously. The data was stored on password-protected data carriers at the Department of General Practice at University Hospital Wuerzburg.

### Ethics

Approval for the study was granted directly by the Ethics Committee at the University of Würzburg (reference number: 20231114 02).

## Results

### Response rate and sample

Of 359 questionnaires analysed, 158 were completed by students admitted through the Bavarian rural doctor quota (44.0%) and 201 by students in the control group (56.0%). In relation to 430 students admitted via the RDQ in Bavaria until the end of the survey, the response rate for RDQ participants was 36.7%. Table 1 shows an overview of the demographic characteristics of the students in comparison.

**Table 1:** Study sample characteristics of both groups

Variable	RDQs	Non-RDQs	*p*
Age in years: *M* (*SD*)	25.15 (3.67)	23.7 (3.31)	< 0.01
Gender: female	93 (58.9%)	161 (80.1%)	< 0.01
male	64 (40.5%)	39 (19.4%)	< 0.01
divers	1 (0.6%)	1 (0.05%)	1
Current semester^*^: *M (SD)*	4.70 (2.24)	4.97 (2.29)	0.272
Begin of medical studies (in full years): *M (SD)*	2.56 (1.20)	2.71 (1.12)	0.235
Vocational training (thereof completed)	63.3.% (31.3%)	24.9% (43.1%)	< 0.01 (0.13)
Other studies (thereof completed)	25.3% (19.7%)	15.4% (12.5%)	0.03 (0.28)
Children	6 (3.8%)	3 (1.9%)	0.29

Medical faculty	**n**	**n**	**sum**
Wuerzburg	35	121	156
Munich	55	24	79
Erlangen	41	29	70
Regensburg	21	14	35
Augsburg°	6	12	18
Not specified	0	1	1
Total	158	201	359

*RDQs* Rural Doctor Quota students, *Non-RDQs* other students with different study admission than RDQ

^*^semester = half an academic year (6 month)

°a new model study program for medicine was established in October 2019

### Attitudes towards the rural doctor quota

RDQ students expressed generally positive attitudes toward pursuing general practice in rural area (for an overview, see Table 2; for specific quotes, see Appendix B). They reported both interest in general practice independent of the contractual service requirement and pride in the prospect of becoming a rural GP, indicating a predominantly favourable orientation toward rural primary care.

**Table 2:** Attitudes towards the rural doctor quota by RDQs students. Agreement with statement on likert scale (1 = “does not apply at all”, 5 = “applies completely”)

Categories and items	M (SD)
Positive orientation towards rural general practice	
- I would be interested in general practice even without a contractual obligation.	3.96 (1.17)
- I am proud to become a general practitioner in a rural area.	3.92 (1.10)
Quota admission as only perceived admission option	
- Without the rural doctor quota, I would not be able to study medicine.	3.90 (1.24)
- Due to the abolition of the waiting period, I opted for the country doctor quota in order to obtain a place at university.	3.22 (1.67)
- I would not commit myself contractually to a specialty again during my studies.	2.37 (1.29)
Circumvention or exit from rural service obligations	
- I could imagine buying myself out of my contractual obligations	2.34 (1.35)
- I could imagine taking legal action to free myself from my contractual obligations.	2.17 (1.31)
- After fulfilling my contractual obligations, I plan to work in a different field.	2.18 (1.19)
Social perception of the rural doctor quota	
- I feel that I have taken a place away from someone else.	2.62 (1.43)
- I avoid telling others that I am studying under the rural doctor quota.	1.17 (0.59)

Regarding reasons for entering the rural doctor quota, many participants perceived the programme as an important or necessary pathway to obtain admission to medical school (see also Quotes 1–2). RDQ students indicated that their decision to apply through the quota was influenced by the removal of the ‘waiting-time quota’ (a German medical school admissions pathway that allocated a proportion of study places to applicants based on the number of years they had been on the waiting list after finishing secondary school; it was abolished in 2020, followed by a three-year transitional arrangement). At the same time, only limited regret was expressed about having committed to a specialty obligation during medical studies.

Intentions to circumvent or withdraw from the contractual rural service requirements were generally low. Participants rarely indicated that they would consider ‘buying out’ of the contract obligations (i.e. to pay the contractual penalty of €250.000), pursuing legal action to avoid the obligation, or planning to leave rural GP immediately after fulfilling the required service period of 10 years.

Perceptions of social stigma or discomfort associated with studying under the rural doctor quota were moderate to low. Fewer RDQ students felt that they had ‘taken a university place away’ from others or that they preferred not to disclose their participation in the quota programme.

The perspective of Non-RDQ students on the rural doctor quota was also assessed (see Appendix B for quotes and Appendix C for statistical details). Attitudes were overall positive: Non-RDQ students generally expressed moderate to low perceptions and assumptions regarding the RDQ students (see also Quotes 9–10). On average, they only partly agreed with the statement that RDQ students prefer not to disclose how they were admitted to medical school. Assumptions that some RDQ students might attempt to buy out or legally challenge their contractual service obligations were moderate. In contrast, agreement was strongest with the statement that some RDQ students had only committed to the quota in order to gain admission to medical school.

Perceptions of unfairness were low; Non-RQD students largely disagreed with the statement that RDQ students take university places away from others. However, free-text responses revealed that some Non-RQD students perceive the RDQ as an unfair system that could reinforce social injustices by giving students from a higher socio-economic background the opportunity to buy out of their contractual service obligations (Quotes 11–12).

### Burdens related to the quota’s formal obligations

RDQ students reported varying levels of stress associated with the contractual obligations of the rural doctor quota (see Table 2 for average stress rating across all RDQ students).

The biggest stress factor was the uncertainty about which regions would be considered underserved at the time of completing further training, i.e., in which region they will have to work after completing specialist training. Other notable stressors included the ten-year commitment, which most likely had an impact on career planning and life decisions (Quote 5). The restriction to a specific medical specialty and the geographical restriction to underserved areas were considered relatively neutral. One participant commented on the specialty restriction, that in his opinion most RDQ students begin their studies with an optimistic attitude even when encountering the risk of not particularly being enthusiastic about either general medicine or internal medicine or having a different desired field of study (Quote 7). When asked which specialty they would choose without the contractual obligation, 26.2% chose General Practice, 51.0% were undecided, and the remaining 26.1% would choose another specialty. The most frequently mentioned alternatives within this subcategory were internal medicine (52.6%, which is included within the RDQ), followed by anaesthesia (43.1%), paediatrics (28,5%), neurology/ psychiatry (22.4%), and gynaecology (13.8%).

Stress was lower for the restriction to the federal state of Bavaria and the assignment to work in a rural region. For instance, geographical assignment was viewed as unproblematic due to previous experiences with living in rural areas (also see Quote 3). In contrast, a fellow student considered to drop out of university due to the conditions of the rural doctor quota and the accompanying restriction of free choice (Quote 6). In the free text section of the survey, one student added that the uncertainty regarding the allocation process itself was the biggest source of stress (Quote 4).

Overall, the perceived average stress decreased from obligations related to future definition of underserved areas and regional uncertainty to those concerning general rural placement.

### Financial strains as an indirectly related burden associated with rural Doctor quota admission

Regarding the funding of their studies, RDQ students experience greater work-related strains and financial burdens. A two-sample test for equality of proportions examined structural differences in how RDQ and Non-RDQ students finance their studies (see Table 3 and Appendix D).

**Table 3:** Financial situation and workload of RDQ students vs. Non-RDQ students

Aspect of study funding	RDQ students (*n* = 158)	Non-RDQ students (*n* = 201)	*p*
Reliance on parental/relative support	72%	85%	0.004
Reliance on partner support	14%	2%	< 0.001
Employment in completed vocational training	53%	19%	< 0.001
Employment in side jobs	28%	49%	< 0.001
Employment during lecture period	68%	53%	0.008
Employment during lecture-free period	72%	55%	0.002

**Self-assessment by students**	*M* (*SD*)	*M* (*SD*)	*p*
Weekly work hours during lectures	10.36 (4.62)	8.12 (5.26)	0.002
Weekly work hours during lecture-free periods	20.37 (12.34)	15.09 (11.29)	0.002
Financed living expenses independently before studies	3.90 (1.49)	2.33 (1.60)	< 0.001
Must work to cover living costs	4.17 (1.26)	3.53 (1.45)	0.004
Regularly neglect studies due to work	2.67 (1.32)	2.17 (1.18)	0.032
Living situation allows concentration on studies	3.82 (1.00)	4.22 (0.90)	< 0.001

Eight sources of income were assessed: RDQ students relied less on parental/relative, more on partner support, and were more likely employed in jobs requiring completed vocational training, whereas Non-RDQ students more often held typical student side jobs. No significant differences were observed for federal student financial aids (both dependent and independent from parents’ income), scholarships, or other income sources (see Appendix E). Some free-text responses pointed out that RDQ students are excluded from financial support under some scholarship programmes due to their admission via the RDQ (Quote 8; also see discussion). RDQ students were also significantly more likely to work alongside their studies. More RDQ students reported employment during both the lecture period and the lecture-free period. They stated to work more hours per week on average, both during lectures and lecture-free periods. Moreover, RDQ students reported having already financed their living expenses independently before starting their studies and described financing their studies as more challenging. They agreed more strongly that they must work during their studies to cover living costs and that they regularly neglect their studies due to work obligations. In contrast, Non-RDQ students more often reported that their living situation allows them to concentrate on their studies.

## Discussion

This study provides the first cross-site description of the situation of students admitted through the rural doctor quota at all Bavarian medical faculties, offering insights into their attitudes, perceived burdens, and financial challenges compared to regularly admitted medical students.

### Attitudes toward the rural Doctor quota and its social perception

We found that students admitted via the rural doctor quota displayed an overall positive orientation towards rural general practice, with high levels of intrinsic interest beyond contractual obligation and a considerable degree of professional identification with the role of a rural GP. At the same time, many participants perceived the quota as an essential pathway into medical school. These results align with international evidence showing positive professional attitudes and strategic motivations among participants in bonded medical programmes and rural track admissions [21, 22]. In our study, regret over entering a binding commitment remained limited. Importantly, intentions to evade contractual obligations or prematurely discontinue the planned activity were reported to be low. Future research needs to examine whether these favourable self-reported intentions translate into long-term rural retention rates, as there are no results on actual career outcomes yet for RDQ students due to the short duration of the RDQ to this day.

Social stigma associated with studying under the rural doctor quota was reported generally low among students: most RDQ reported little discomfort in disclosing their admission pathway. Similarly, non-RDQ students expressed largely positive or neutral attitudes toward RDQ participants, with low levels of perceived unfairness. However, underlying tensions remained evident in the free-text responses: the perception that RDQ participation may be primarily motivated by strategic access to medical school, as well as concerns that wealthier students might be able to ‘buy themselves out’ of contractual obligations, point to latent normative conflicts about social fairness. This reflects broader debates in the literature on affirmative or targeted admission pathways, where similar patterns of initial acceptance combined with persistent concerns about justice and legitimacy have been reported [23–26]. Because German medical school admissions remain highly selective - still mainly on high school grades - clear and transparent implementation is essential to prevent social delegitimisation (for more details on possible approaches, see e.g. Anachebe et al. [27] and George et al. [28]).

### Frequent stresses and implications for the design of university support programmes

RDQ students face distinct study-related challenges due to structural and individual circumstances. Older students often find it difficult to re-enter the intensive learning environment of medical studies [29–31], with a focus heavily on memorising extensive fundamentals of natural science in German curricula in the first two years [32]. Other studies indicate that older students often have more commitments in terms of family and relationships, which are associated with a greater investment of time and financial obligations [33–35].

RDQ students reported frequent work-study conflicts and financial burdens, causing high stress that often negatively affects academic performance [36]. As many RDQ students are older and have completed prior medical-related vocational training, they often face reduced access to government grants and low-interest student loans, which are primarily designed for students enrolling directly after high school [37]. Even more, overlapping contractual obligations exclude RDQ students from scholarships, such as the Bavarian Country Doctor Scholarship [38]. This stands in contrast with study results showing that financial support measures within programme design is crucial for equity and for achieving long-term retention in rural practice [39].

The contractual obligations within the framework of the quota place additional pressure on RDQ students: uncertainties, e.g. the vague definition of possible working regions for compulsory rural medical practice, proved to be significant stress factors. The ten-year service commitment adds further perceived burden, influencing personal development and family decisions [16]. However, good compatibility between work with family and private life is an important factor in the decision to become a GP [40–42]. While specialty restrictions were less concerning, some students perceived them as limitations on professional autonomy. Extending the rural doctor quota to specialties like paediatrics appears beneficial: it has been implemented in some federal states and provides greater flexibility for adjusting career preferences during training [14]. A challenge remains as many uncertainties relate to factors that cannot be reliably predicted due to future dynamics within the healthcare system. In this context, regularly updated information may support transparency and programme credibility rather than resolve uncertainty about the specific workplace. Complementary measures, including clearer legal definitions, early communication of allocation procedures, and structured counselling on long-term career and life planning, may further reduce perceived uncertainty.In summary, simply obtaining a place at university is not enough: having less time to study due to working alongside studies and facing high levels of stress are factors that are closely associated with impaired academic performance, delayed study progression, and higher dropout risk [43–45]. Moreover, uncertainty regarding academic expectations, examinations, and future career pathways may further exacerbate psychosocial strain among RDQ students. Without targeted institutional support, these cumulative disadvantages threaten not only individual academic success but also the overarching goal of the RDQ programme to sustainably strengthen the rural medical workforce. Consequently, comprehensive university-based programmes that combine curricular structuring, mentoring, and psychosocial support are essential to mitigate these risks and enable RDQ students to successfully complete their studies. In order to demonstrate the importance of university-based programmes, further parts of the survey are currently being evaluated, which we intend to present in a subsequent publication, focusing on the academic achievements of RDQ students and their support needs.

An example of such a programme in Germany is ‘HeLaMed’ (‘Hesse – Countryside – Medicine‘’) that has been implemented at all medical faculties in Hesse and in which all RDQ students in Hesse are required to participate [15, 46, 47]. Hesse is the only federal state in Germany that established a special curriculum for RDQ students *by law* - not voluntary as in other medical faculties - and provides necessary resources for universities and for accompanying research [48]. ‘HeLaMed’ integrates mentoring, early placements in rural general practices, thematic seminars (e.g., digitalisation, interprofessional collaboration), and network-building throughout the course of study, preparing students practically, socially, and professionally for a career as rural GPs. An example of a voluntary support programme is the BeLA (‘Beste Landpartie Allgemeinmedizin’ – best rural outing GP) programme in Bavaria, which offers an in-depth support programme for students interested in rural GP [49].

National and international evidence underscores the effectiveness of this approach. Physicians with a rural background, or those who gained rural clinical experience during training, are far more likely to practise in rural areas, with early and longitudinal placements showing the strongest impact [50, 51]. Programmes such as Australia’s Remote Vocational Training Scheme and Canada’s Physician Shortage Area Program combine practical rural experience, supervision, and social support, achieving high completion rates and long-term retention in underserved regions [52–54]. Systematic reviews further highlight that multi-component interventions - including preferential admission for students with rural backgrounds, hands-on rural training, and extended placements - are particularly successful [55].

### Strength and limitations

To our knowledge, this is the first cross-location study on the study situation of RDQ students in Germany. The use of a very detailed questionnaire in combination with the analysis of free text answers provides wide-ranging insight into a relatively underexplored population, whose motivations and perceptions are directly relevant for rural workforce planning. The online format allowed for broad reach and efficient data collection, enabling the inclusion of students from different institutions and study stages. Several limitations should be acknowledged: Participation was voluntary, which may introduce self-selection bias, as students with stronger opinions about rural practice or the quota may have been more likely to respond. The cross-sectional design captures attitudes at a single point in time and does not allow for conclusions about how motivations or perceptions may change throughout medical training. Additionally, the study relied on self-reported data, which may be influenced by social desirability or recall bias. The qualitative content analysis of free-text responses was conducted by a single author, without independent double coding, which may limit interpretative reliability. Moreover, qualitative data were collected through open-ended survey questions rather than in-depth interviews, limiting opportunities for probing, clarification, and follow-up questions. Finally, while the findings are informative for the Bavarian context, they may not be fully generalisable to other federal states with different quota structures or admission procedures.

## Conclusions

RDQ students face particular challenges. Beyond allocating university places, universities and policymakers should offer structured curricula that include mentoring, early and regular internships in rural areas, academic counselling, psychosocial support to improve both the recruitment and long-term retention of young doctors in rural areas, next to a targeted financial support at federal or state level. Clear and updated information about underserved areas, as well as transparent internship and scholarship criteria, are essential to reduce uncertainty and inequality to build trust in the program, and to support individual career planning. Embedding the RDQ quota programme in a broader educational and institutional strategy, including ongoing evaluation, is essential to link the students’ initial motivation to the long-term objectives of the rural doctor quota. International findings underscore that such a multi-layered approach is crucial for long-term retention and the sustainable provision of rural healthcare.

## Supplementary Information

Supplementary Material 1.

## Data Availability

The datasets used and analysed in the present study are available from the corresponding author upon reasonable request.

## Abbreviations

BeLA: Beste Landpartie Allgemeinmedizin (German for: Best rural outing General Practice; voluntary Bavarian support programme for students)
CHERRIES: Checklist for reporting results of internet E-surveys
EGDPR: European General Data Protection Regulation
FD: Family doctor
GP: General Practice
GPs: General Practitioner
HeLaMed: Hessen – Land – Medizin (German for: Hesse – Countryside – Medicine; obligatory programme for RDQ students in Hesse)
LAQ: Landarztquote (German for: rural doctor quota)
LGL: Bayerisches Landesamt für Gesundheit und Lebensmittelsicherheit (German for: Bavarian State Office for Health and Food Safety)
Non-RDQ students: Students who were not admitted to medical studies via the rural doctor quota
RDQ: Rural Doctor Quota
RDQ students: Students admitted to medical studies via the rural doctor quota
