# Supplementary material for: Rural admission quota in Germany: students’ attitudes, challenges and the need for support programmes
Source: BMC Med Educ. 2026 Feb 11;26:340. doi: 10.1186/s12909-026-08776-w (PMC12930864; doi:10.1186/s12909-026-08776-w)
Supplement: Supplementary file 1 — Supplementary Material 1. [file 12909_2026_8776_MOESM1_ESM.pdf]

Appendix A: Survey excerpt (items relevant to the current study)

The option "not specified" (original German: “keine Angabe”) was available for most scale-based items; answering questions was voluntary. For single and multiple-choice items, all options are displayed below the respective question against a grey background. For Likert scales, the instructions and scale labels are presented with orange background before listing the items. Section titles and introductory remarks are included for completeness and are displayed with a blue background.

Nr.	Type	German (original)	English (translated)
**2**	**Section title**	**Demografische Angaben**	**Demographic information**
2.1	Open question	Geburtsjahr (z. B. 1998)	Year of birth (e. g. 1998)
2.2	Single choice	Geschlecht	Gender
	Option 1	Weiblich	Female
	Option 2	Männlich	Male
	Option 3	Divers	Diverse
2.3	Single choice	Familienstand	Marital status
	Option 1	Ledig	Single
	Option 2	In Partnerschaft	In a relationship
	Option 3	Verheiratet	Married
	Option 4	Sonstiges	Other
2.4	Single choice	Wie viele Kinder hast du?	How many children do you have?
	Option 1	0	0
	Option 2	1	1
	Option 3	2	2
	Option 4	3	3
	Option 5	Mehr als 3	More than 3
2.5	Single choice	Welche Universität besuchst du?	Which university do you attend?
	Option 1	Würzburg	Würzburg
	Option 2	Erlangen	Erlangen
	Option 3	Augsburg	Augsburg
	Option 4	LMU München	LMU Munich
	Option 5	TU München	TU Munich
2.6	Single choice	Nimmst du am Programm „Beste Landpartie - Allgemeinmedizin“ teil?	Are you participating in the "Best rural outing – General Practice” program?
	Option 1	Ja	Yes
	Option 2	Nein	No
2.7	Open question	In welchem Fachsemester bist du?	Which semester are you in?

2.8	Single choice	Wann hast du dein Studium begonnen?	When did you start your studies?
	Option 1	SS2020 oder früher	SS2020 or earlier
	Option 2	WS2020/21	WS2020/21
	Option 3	SS2021	SS2021
	Option 4	WS2021/22	WS2021/22
	Option 5	SS2022	SS2022
	Option 6	WS2022/23	WS2022/23
	Option 7	SS2023	SS2023
	Option 8	WS2023/24	WS2023/24
	Option 9	SS2024	SS2024
2.9	Single choice	Über welches Verfahren hast du deinen Studienplatz erhalten?	Through which procedure did you receive your study place?
	Option 1	Abiturbestenquote	Top Abitur grade quota
	Option 2	Auswahlverfahren der Hochschulen	University selection procedure
	Option 3	Zusätzliche Eignungsquote	Additional aptitude quota
	Option 4	Wartesemester	Waiting semesters
	Option 5	Landarztquote	Rural doctor quota
	Option 6	ÖGD-Quote	Public health service quota
	Option 7	Studiumsberechtigung über die Bundeswehr	University eligibility via military
	Option 8	Quote der Nicht-EU-Ausländer	Non-EU quota
	Option 9	Weiß ich nicht	Don’t know
	Option 10	Sonstiges	Other
2.10	Open question	Sonstiges: Bitte frei eintragen.	Other: Please enter freely.
2.11	Single choice	Wodurch hast du die Hochschulzugangsberechtigung für das Medizinstudium erlangt?	How did you obtain your higher education entrance qualification for medical school?
	Option 1	Allgemeine Hochschulreife	General higher education entrance qualification (Abitur)
	Option 2	Fachgebundene Hochschulreife	Subject-specific higher education entrance qualification
	Option 3	Hochschulzugang durch berufliche Aufstiegsfortbildung	University entrance via advanced vocational training
	Option 4	Hochschulzugang mit fachlich entsprechender Berufsausbildung	University entrance with a relevant vocational qualification
	Option 5	Hochschulzugang mit fachfremder Berufsausbildung und Zugangsprüfung	University entrance with non-related vocational training and entrance examination
	Option 6	Vergleichbare Qualifikation im Ausland	Comparable qualification obtained abroad

2.12	Open question	Wann hast du deine Hochschulzugangsberechtigung erlangt? (Jahreszahl)	When did you obtain your higher education entrance qualification? (year)
2.13	Single choice	In welchem Bundesland hast du diese erlangt?	In which federal state did you obtain it?
	Option 1	Bayern	Bavaria
	Option 2	Baden-Württemberg	Baden-Württemberg
	Option 3	Hessen	Hesse
	Option 4	Thüringen	Thuringia
	Option 5	Sachsen	Saxony
	Option 6	Rheinland-Pfalz	Rhineland-Palatinate
	Option 7	Saarland	Saarland
	Option 8	Nordrhein-Westfalen	North Rhine-Westphalia
	Option 9	Niedersachsen	Lower Saxony
	Option 10	Bremen	Bremen
	Option 11	Sachsen-Anhalt	Saxony-Anhalt
	Option 12	Brandenburg	Brandenburg
	Option 13	Berlin	Berlin
	Option 14	Hamburg	Hamburg
	Option 15	Schleswig-Holstein	Schleswig-Holstein
	Option 16	Mecklenburg-Vorpommern	Mecklenburg-Western Pomerania
2.14	Single choice	Hast du nach deiner schulischen Ausbildung direkt das Studium aufgenommen?	Did you start your medical studies directly after finishing school?
	Option 1	ja	yes
	Option 2	nein	no
2.15	Multiple choice	Welche Tätigkeit hast du zwischen Schulabschluss und Aufnahme des Medizinstudiums verfolgt?	Which activity did you pursue between finishing school and starting medical school?
	Instruction	(mehrere Angaben möglich)	(multiple answers possible)
	Option 1	Berufsausbildung	Vocational training
	Option 2	Anderes Studium	Other degree program
	Option 3	Erwerbstätigkeit	Employment
	Option 4	Auslandsaufenthalt	Stay abroad
	Option 5	Freiwilligendienst	Voluntary service
	Option 6	Ehrenamtliche Tätigkeit	Voluntary work
	Option 7	Sonstiges	Other
2.16	Open question	Sonstiges	Other
2.17	Single choice	Wie viele Studiengänge hast du vor dem Medizinstudium begonnen?	How many degree programs did you start before medical school?
	Option 1	1	1

	Option 2	2	2
	Option 3	3	3
	Option 4	4	4
	Option 5	>4	>4
2.18	Open question	Welches Studium hast du begonnen?	Which degree program did you start?
2.19	Single choice	Hast du das Studium abgeschlossen?	Did you complete this degree program?
	Option 1	ja	yes
	Option 2	nein	no
2.20	Open question	In welchem Jahr hast du das Studium abgeschlossen?	In which year did you complete this degree program?
2.21	Open question	Wie viele Fachsemester hast du in diesem Studiengang studiert?	How many subject-specific semesters did you study in this degree program?
2.22	Open question	Welches Studium hast du dann begonnen?	Which degree program did you start next?
2.23	Single choice	Hast du das Studium abgeschlossen?	Did you complete this degree program?
	Option 1	ja	yes
	Option 2	nein	no
2.24	Open question	In welchem Jahr hast du das Studium abgeschlossen?	In which year did you complete this degree program?
2.25	Open question	Wie viele Fachsemester hast du in diesem Studiengang studiert?	How many subject-specific semesters did you study in this degree program?
2.26	Open question	Welches Studium hast du dann begonnen?	Which degree program did you start then?
2.27	Single choice	Hast du das Studium abgeschlossen?	Did you complete this degree program?
	Option 1	ja	yes
	Option 2	nein	no
2.28	Open question	In welchem Jahr hast du das Studium abgeschlossen?	In which year did you complete this degree program?
2.29	Open question	Wie viele Fachsemester hast du in diesem Studiengang studiert?	How many subject-specific semesters did you study in this degree program?
2.30	Open question	Welches Studium hast du dann begonnen?	Which degree program did you start then?
2.31	Single choice	Hast du das Studium abgeschlossen?	Did you complete this degree program?
	Option 1	ja	yes
	Option 2	nein	no
2.32	Open question	In welchem Jahr hast du das Studium abgeschlossen?	In which year did you complete this degree program?
2.33	Open question	Wie viele Fachsemester hast du in diesem Studiengang studiert?	How many subject-specific semesters did you study in this degree program?

2.34	Open question	Welche Studiengänge hast du noch begonnen?	Which other degree programs did you start?
2.35	Multiple choice	Welche Ausbildung hast du begonnen? (mehrere Angaben möglich)	Which vocational training did you start? (multiple answers possible)
	Option 1	Rettungssanitäterin	Emergency medical technician
	Option 2	Notfallsanitäterin	Paramedic
	Option 3	Pflegefachmann/-frau bzw. Gesundheits- und (Kinder)krankenpflegerin	Nursing professional / health and (pediatric) nurse
	Option 4	Hebamme	Midwife
	Option 5	MTL/MTLA	Medical technical laboratory assistant
	Option 6	Physiotherapeutin	Physiotherapist
	Option 7	MTR/MTRA	Medical technical radiology assistant
	Option 8	MFA	Medical assistant
	Option 9	Sonstige	Other
2.36	Open question	Sonstige	Other
2.37	Single choice	Hast du die Ausbildung als Rettungssanitäter*in abgeschlossen?	Did you complete the training as an emergency medical technician?
	Option 1	ja	yes
	Option 2	nein	no
2.39	Single choice	Hast du die Ausbildung als Notfallsanitäter*in abgeschlossen?	Did you complete the training as a paramedic?
	Option 1	ja	yes
	Option 2	nein	no
2.41	Single choice	Hast du die Ausbildung als Pflegefachmann/-frau bzw. Gesundheits- und (Kinder)krankenpfleger*in abgeschlossen?	Did you complete the training as a nursing professional / health and (pediatric) nurse?
	Option 1	ja	yes
	Option 2	nein	no
2.43	Single choice	Hast du die Ausbildung als Hebamme abgeschlossen?	Did you complete the training as a midwife?
	Option 1	ja	yes
	Option 2	nein	no
2.45	Single choice	Hast du die Ausbildung als MTL/MTLA abgeschlossen?	Did you complete the training as a medical technical laboratory assistant (MTL/MTLA)?
	Option 1	ja	yes
	Option 2	nein	no
2.47	Single choice	Hast du die Ausbildung als Physiotherapeut*in abgeschlossen?	Did you complete the training as a physiotherapist?

	Option 1	ja	yes
	Option 2	nein	no
2.49	Single choice	Hast du die Ausbildung als MTR/MTRA abgeschlossen?	Did you complete the training as a medical technical radiology assistant (MTR/MTRA)?
	Option 1	ja	yes
	Option 2	nein	no
2.51	Single choice	Hast du die Ausbildung als MFA abgeschlossen?	Did you complete the training as a medical assistant (MFA)?
	Option 1	ja	yes
	Option 2	nein	no
2.53	Single choice	Hast du diese Ausbildung abgeschlossen?	Did you complete this other training?
	Option 1	ja	yes
	Option 2	nein	no
3	Section title	Situation/Anforderungen außerhalb des Studiums	Situation / Requirements outside the course of study
	Instruction	Im Folgenden werden dir Fragen über die Rahmenbedingungen deines Studiums und den Studienalltag gestellt.<br>**Finanzierung und Arbeitstätigkeit neben dem Studium:**<br>Wie finanzierst du dein Studium?	Below, you will be asked questions about the general conditions of your studies and everyday student life.<br>**Financing and working alongside your studies:**<br>How do you finance your studies?
3.1	Single choice	Elternabhängiges BAföG?	Subsidized student loan dependent on parents' income?
	Option 1	ja	yes
	Option 2	nein	no
3.2	Open question	Bitte schätze den prozentualen Anteil des elternabhängigen BAföG an deiner Finanzierung (0–100).	Please estimate the percentage share of parental-income based student loan in your financing (0–100).
3.3	Single choice	Elternunabhängiges BAföG?	Student loan independent of parents' income?
	Option 1	ja	yes
	Option 2	nein	no
3.4	Open question	Bitte schätze den prozentualen Anteil des elternunabhängigen BAföG an deiner Finanzierung (0–100).	Please estimate the percentage share of parental-independent student loan in your financing (0–100).
3.5	Single choice	Ein oder mehrere Stipendien?	One or more scholarships?
	Option 1	ja	yes
	Option 2	nein	no

3.6	Open question	Bitte schätze den prozentualen Anteil des Stipendiums/aller Stipendien an deiner Finanzierung (0–100).	Please estimate the percentage share of your scholarship(s) in your financing (0–100).
3.7	Single choice	Eltern/Angehörige?	Parents / relatives?
	Option 1	ja	yes
	Option 2	nein	no
3.8	Open question	Bitte schätze den prozentualen Anteil an deiner Finanzierung durch Eltern/Angehörige (0–100).	Please estimate the percentage share of your financing provided by parents/relatives (0–100).
3.9	Single choice	Meine Partnerin?	My partner?
	Option 1	ja	yes
	Option 2	nein	no
3.10	Open question	Bitte schätze den prozentualen Anteil an deiner Finanzierung durch deine Partnerin (0–100).	Please estimate the share of your financing contributed by your partner (0–100).
3.11	Single choice	Eine Erwerbstätigkeit in Beruf mit abgeschlossener Ausbildung?	Employment in a profession with completed vocational training?
	Option 1	ja	yes
	Option 2	nein	no
3.12	Open question	Bitte schätze den prozentualen Anteil der Erwerbstätigkeit in deinem Beruf an deiner Finanzierung (0–100).	Please estimate the percentage share of employment in your trained profession in your financing (0–100).
3.13	Single choice	Einen Nebenjob (z. B. Gastronomie)?	A side job (e.g., in gastronomy)?
	Option 1	ja	yes
	Option 2	nein	no
3.14	Open question	Bitte schätze den prozentualen Anteil des Nebenjobs an deiner Finanzierung (0–100).	Please estimate the percentage share of your side job in your financing (0–100).
3.15	Single choice	Sonstige Einkünfte?	Other sources of income?
	Option 1	ja	yes
	Option 2	nein	no
3.16	Open question	Bitte schätze den prozentualen Anteil der sonstigen Einkünfte an deiner Finanzierung (0–100).	Please estimate the percentage share of other income sources in your financing (0–100).
3.17	Open question	Sonstige Einkünfte (bitte angeben):	Other income (please specify):
3.18	Single choice	Wie ist deine aktuelle Wohnsituation?	What is your current housing situation?
	Option 1	allein	living alone
	Option 2	Wohngemeinschaft	shared apartment (WG)
	Option 3	Studierendenwohnheim	student dormitory
	Option 4	bei Eltern/Angehörigen	living with parents/relatives

	Option 5	mit Partner*in	with partner
	Option 6	Sonstige	other
3.19	Open question	Sonstige (bitte angeben):	Other (please specify):
3.20	Single choice	Konntest du deinen Lebensunterhalt vor dem Studium eigenständig finanzieren?	Were you able to support yourself financially before starting your studies?
	Option 1	ja	yes
	Option 2	nein	no
3.21	Single choice	Arbeitest du in der Vorlesungszeit?	Do you work during the lecture period?
	Option 1	ja	yes
	Option 2	nein	no
3.22	Open question	Wie viele Wochenstunden arbeitest du in der Vorlesungszeit durchschnittlich?	How many hours per week do you work during the lecture period on average?
3.23	Single choice	Arbeitest du in der vorlesungsfreien Zeit?	Do you work during the semester break?
	Option 1	ja	yes
	Option 2	nein	no
3.24	Open question	Wie viele Wochenstunden arbeitest du in der vorlesungsfreien Zeit durchschnittlich?	How many hours per week do you work during the semester break on average?
	Introduction for five-step Likert scale items	Gib bitte im Folgenden an, inwieweit die Aussagen auf dich zutreffen.	Please indicate below to what extent the statements apply to you.
	Labelling of scale level 1	Trifft gar nicht zu	Does not apply at all
	Labelling of scale level 5	Trifft voll zu	Applies completely
3.25	Likert scale	Meine Lebenssituation erlaubt mir, mich auf mein Studium zu konzentrieren.	My living situation allows me to focus on my studies.
3.26	Likert scale	Ich fühle mich im Falle einer finanziellen Notfallsituation abgesichert (z. B. Kaution, Auto defekt).	I feel financially secure in case of an emergency (e.g., deposit, car repair).
3.27	Likert scale	Ich kann meine Arbeitszeit grundsätzlich flexibel gestalten.	I can generally organize my working hours flexibly.
3.28	Likert scale	Ich vernachlässige mein Studium regelmäßig aufgrund meiner Arbeitstätigkeit.	I regularly neglect my studies because of my work activities.
3.29	Likert scale	An einem Arbeitstag lerne ich genauso motiviert wie an einem arbeitsfreien Tag.	On a working day, I study as motivated as on a day off.
3.30	Likert scale	Ich muss arbeiten, um mein Leben während des Studiums zu finanzieren.	I have to work to pay for my living expenses during my studies.

3.31	Likert scale	Durch meine Arbeit profitiere ich fachlich für mein Studium.	Through my job, I gain professional benefits for my studies.
6	Section title	Fragen zur Landarztquote	Questions about the rural doctor quota
6.1	Single choice	Kennst du Studierende, die über die Landarztquote studieren?	Do you know students who are studying under the rural doctor quota?
	Option 1	ja	yes
	Option 2	nein	no
6.2	Single choice	Ohne die vertraglichen Verpflichtungen würde ich ein anderes Fachgebiet wählen.	Without the contractual obligations, I would choose another specialty.
	Option 1	ja	yes
	Option 2	nein	no
	Option 3	ich weiß noch nicht	I don't know yet
6.3	Multiple choice	Welches? (mehrfache Auswahl möglich)	Which one(s)? (multiple answers possible)
	Option 1	weiß ich noch nicht	I don't know yet
	Option 2	Anästhesie	Anesthesiology
	Option 3	Chirurgie	Surgery
	Option 4	Innere Medizin	Internal medicine
	Option 5	Pädiatrie	Pediatrics
	Option 6	HNO	ENT (ear, nose, throat)
	Option 7	Psychiatrie	Psychiatry
	Option 8	Neurologie	Neurology
	Option 9	Gynäkologie	Gynecology
	Option 10	Sonstiges	Other
6.4	Open question	Sonstiges	Other
	Introduction for five-step Likert scale items	An den vertraglichen Verpflichtungen finde ich folgende Aspekte belastend:	I find the following aspects of the contractual obligations burdensome:
	Labelling of scale level 1	Gar nicht	Not at all
	Labelling of scale level 5	Sehr	Very
6.5	Likert scale	Örtliche Einschränkung auf unterversorgte Gebiete	Limitation to underserved regions
6.6	Likert scale	Einschränkung des Arbeitsplatzes auf Bayern	Restriction of workplace to Bavaria
6.7	Likert scale	Zehnjährige Verpflichtung	Ten-year obligation
6.8	Likert scale	Facharzteinschränkung	Specialty restriction
6.9	Likert scale	Ländliche Arbeitsregion	Rural work region

6.10	Likert scale	Ungewissheit, welche Regionen bei Abschluss der Facharztausbildung als unterversorgt gelten	Uncertainty about which regions will be considered underserved after specialisation
6.11	Likert scale	Ohne die Landarztquote wäre es mir nicht möglich, Medizin zu studieren.	Without the rural doctor quota, it would not have been possible for me to study medicine.
6.12	Introduction for five-step Likert scale items	Gib bitte im Folgenden an, inwieweit die Aussagen auf dich zutreffen.	Please indicate below to what extent the statements apply to you.
	Labelling of scale level 1	trifft gar nicht zu	Does not apply at all
	Labelling of scale level 5	trifft voll zu	Applies completely
	Likert scale	Ich würde mich auch ohne die vertragliche Verpflichtung für die Allgemeinmedizin interessieren.	I would be interested in general practice even without the contractual obligation.
6.13	Likert scale	Aufgrund der Abschaffung der Wartezeit habe ich die Landarztquote gewählt, um einen Studienplatz zu erhalten.	I chose the rural doctor quota because the waiting-time admission option was abolished.
6.14	Likert scale	Ich würde mich nicht noch einmal vertraglich in meinem Studium für ein Fachgebiet verpflichten.	I would not commit myself contractually to a specialty again during my studies.
6.15	Likert scale	Ich kann mir vorstellen, mich aus meinen vertraglichen Verpflichtungen freizukaufen.	I can imagine buying myself out of my contractual obligations.
6.16	Likert scale	Ich kann mir vorstellen, mich aus meinen vertraglichen Verpflichtungen herauszuklagen.	I can imagine taking legal action to get out of my contractual obligations.
6.17	Likert scale	Nach Erfüllung der vertraglichen Verpflichtungen plane ich, in einem anderen Fachgebiet tätig zu werden.	After fulfilling the contractual obligations, I plan to work in another specialty.
6.18	Likert scale	Ich vermeide es, Anderen zu erzählen, dass ich über die Landarztquote studiere.	I avoid telling others that I am studying under the rural doctor quota.
6.19	Likert scale	Ich habe das Gefühl, Anderen den Studienplatz weggenommen zu haben.	I feel that I have taken away a study place from others.
6.20	Likert scale	Ich bin stolz darauf, Allgemeinmedizinerin in einer ländlichen Region zu werden.	I am proud to become a general practitioner in a rural region.
	Introduction for five-step Likert scale items	Bitte bewerte folgende Aussagen nach deinem subjektiven Empfinden.	Please rate the following statements according to your subjective opinion.

	Labelling of scale level 1	Trifft gar nicht zu	Does not apply at all
	Labelling of scale level 5	Trifft voll zu	Applies completely
6.21	Likert scale	Landarztquoten-Studierende behalten es lieber für sich, wie sie zum Studium zugelassen wurden.	Students admitted through the rural doctor quota prefer not to disclose how they were admitted.
6.24	Likert scale	Ich kann mir vorstellen, dass sich einige Landarztquoten-Studierende aus ihren vertraglichen Verpflichtungen freikaufen.	I can imagine that some rural quota students will buy themselves out of their contractual obligations.
6.25	Likert scale	Ich kann mir vorstellen, dass sich einige Landarztquoten-Studierende aus ihren vertraglichen Verpflichtungen herausklagen.	I can imagine that some rural quota students will try to annul their obligations through legal action.
6.26	Likert scale	Einige Landarztquoten-Studierende haben sich nur für die Quote verpflichtet, um überhaupt Medizin studieren zu können.	Some rural quota students committed to the program just to be able to study medicine at all.
6.27	Likert scale	Ich finde, die Landarztquoten-Studierenden nehmen Anderen die Studienplätze weg.	I think rural quota students take study places away from others.
6.28	Open question	Gibt es noch etwas zur Landarztquote, was du loswerden möchtest?	Is there anything else you would like to say about the rural doctor quota?

Appendix B: Attitudes towards RDQ by both groups, quotes extracted from the free-text responses

RDQ students: Themes and quotes

Main Themes	Quote	Translation into British English	Original wording in German
**Favourable aspects**			
General positive attitudes	1	*"Would study under the RDQ over and over again."*	*„Ich würde immer wieder über die Landarztquote studieren."*
RDQ to gain access to studies	2	*"The RDQ was fortunately the last chance to get a place after waiting for 12 semesters."*	*„Die Landarztquote war glücklicherweise die letzte Chance, nach 12 Wartesemestern einen Studienplatz zu bekommen."*
Familiarity with rural area	3	*"Since I come from the countryside and the area will remain underserved, this commitment is no problem for me."*	*„Da ich vom Land komme und die Region unterversorgt bleiben wird, ist diese Verpflichtung kein Problem für mich."*
**Unfavourable aspects**			
Uncertainty regarding future	4	*"The uncertainty surrounding the future award procedure is causing me more headaches than the commitment itself."*	*„Die Unsicherheit über das zukünftige Vergabeverfahren bereitet mir mehr Kopfzerbrechen als die Verpflichtung selbst."*
	5	*"I find it terrible… that I don't know where I will be allowed to work after specialist training… finishing at 30/31, five years of training, then children… means I will have to reorient myself locally at ~38–40."*	*„Ich finde es schrecklich… dass* ich nicht weiß, wo ich nach meiner Weiterbildung arbeiten *darf… mit 30/31 fertig, fünf* Jahre Weiterbildung, dann *Kinder… bedeutet, dass ich* mich mit ~38–40 komplett neu *orientieren muss."*
Restriction of personal freedom	6	*"Still very often consider dropping out… The fact of being sent to some backwater at the discretion of the authorities feels like serfdom and has nothing to do with freedom."*	*„Überlege immer noch sehr oft, abzubrechen... Die Tatsache, in irgendein Kaff nach Belieben des Amtes abgeschoben zu werden fühlt sich an wie Leibeigenschaft und hat mit Freiheit nichts zu tun."*
Specialist restriction	7	*"Many LAQ students embark on their studies on a 'hope for the best'*	*„Viele LAQ-Studierende beginnen ihr Studium nach dem Motto ‚es wird schon gut gehen'*

		basis… risking not being enthusiastic about general or internal medicine, or having a different desired field."	*und riskieren, weder für Allgemein- noch Innere Medizin begeistert zu sein oder ein anderes Wunschfach zu haben."*
Limited financial support	8	*"I was not eligible for many scholarships because I obtained my place through the RDQ. Financial support would be helpful."*	*„Da ich meinen Studienplatz über die Landarztquote erhalten habe, war ich nicht für viele Stipendien berechtigt. Finanzielle Unterstützung wäre hilfreich."*

Non-RDQ students: Themes and quotes

Main Themes	Quote	Translation into British English	Original wording in German
**Favourable aspects**			
General positive attitudes	9	*"I think it's a very good win-win situation for Germany and for people who want to become doctors!"(Non-RDQ)*	*„Ich finde es ist eine sehr gute Win-Win Situation für Deutschland und die Personen, die gerne Arzt/Ärztin werden wollen!" (Nicht-LAQ)*
	10	*"Is a great option if your A-levels aren't good enough." (Non-RDQ)*	*„Ist eine tolle Option, wenn das Abitur nicht gut genug ist." (Nicht-LAQ)*
**Unfavourable aspects**			
Reinforcement of social injustice	11	*"This [RDQ] does not make studying fair for all social classes. Rich children of doctors can buy their way out and sue. Financially disadvantaged fellow students do not have this option. This involuntarily creates a kind of paid private education." (Non-RDQ)*	*„[Landarztquote] macht das Studium nicht fair für alle sozialen Schichten. Reiche Arztkinder können sich freikaufen und -klagen. Finanziell schlechter gestellte Kommilitonen haben diese Möglichkeit nicht. Dadurch entsteht unfreiwillig eine Art bezahltes Privatstudium." (Nicht-LAQ)*
	12	*"I am critical of taking advantage of this opportunity despite having other career aspirations. Precisely because many want to sue their way out of it, thereby defeating the purpose of attracting rural doctors." (Non-RDQ)*	*„Nutzung der Möglichkeit trotz anderer Karrierevorstellungen sehe ich kritisch. Gerade weil viele sich gerichtlich freiklagen wollen und somit der Sinn Landärzte zu gewinnen verloren geht." (Nicht-LAQ)*

Appendix C: Non-RDQ students' statements about RDQ students.

Response to Likert scale: 1 = “does not apply at all”, 5 = “applies completely”. *M* = Mean, *SD* = Standard deviation. Statistical analysis: two-sided independent samples t-tests (significance level of 0.05).

**Item**	***M***	***SD***
RDQ students prefer not to disclose how they were admitted to medical school.<br>*Landarztquoten-Studierende behalten es lieber für sich, wie sie zum Studium zugelassen wurden.*	2.48	1.25
Some RDQ students might attempt to buy out their contractual service obligations<br>*Ich vermute, dass sich einige Landarztquoten-Studierende aus ihren vertraglichen Verpflichtungen freikaufen.*	3.10	1.35
Some RDQ students might legally challenge their contractual service obligations.<br>*Ich vermute, dass sich einige Landarztquoten-Studierende aus ihren vertraglichen Verpflichtungen herausklagen.*	3.04	1.39
Some RDQ students had only committed to the quota to gain admission to medical school<br>*Einige Landarztquoten-Studierende haben sich nur für die Quote verpflichtet, um überhaupt Medizin studieren zu können.*	4.28	0.87
RDQ students take university places away from others<br>*Ich finde, die Landarztquoten-Studierenden nehmen Anderen die Studienplätze weg.*	1.29	0.71

Appendix D: Financial situation

Response to Likert scale: 1 = “does not apply at all”, 5 = “applies completely”. *M* = Mean, *SD* = Standard deviation. Statistical analysis: two-sided independent samples t-tests (significance level of 0.05; ** = <.01).

**Item**	**Group**	***M***	***SD***	***p***
On a workday, I am just as motivated to study as on a day off.	RDQ	2.18	1.25	.520
An einem Arbeitstag lerne ich genauso motiviert wie an einem arbeitsfreien Tag.	Non-RDQ	2.29	1.18	
Through my work, I gain professional benefits for my studies.	RDQ	3.88	1.24	.064
Durch meine Arbeit profitiere ich fachlich für mein Studium.	Non-RDQ	3.55	1.42	
I feel financially secure in case of an emergency (e.g., paying a deposit, car breakdown).	RDQ	3.45	1.41	.286
Ich fühle mich im Falle einer finanziellen Notfallsituation abgesichert (z.B. Kaution hinterlegen, Auto defekt).	Non-RDQ	3.60	1.34	
I can generally arrange my working hours flexibly.	RDQ	4.25	0.95	.397
Ich kann meine Arbeitszeit grundsätzlich flexibel gestalten.	Non-RDQ	4.15	0.99	
I was already able to finance my living expenses independently before starting my studies.	RDQ	3.90	1.49	<.001**
Ich konnte meinen Lebensunterhalt vor dem Studium bereits eigenständig finanzieren.	Non-RDQ	2.33	1.60	
I have to work to finance my life during my studies.	RDQ	4.17	1.26	<.001**
Ich muss arbeiten, um mein Leben während des Studiums zu finanzieren.	Non-RDQ	3.53	1.45	
I regularly neglect my studies because of my work.	RDQ	2.67	1.32	.004**
Ich vernachlässige mein Studium regelmäßig aufgrund meiner Arbeitstätigkeit.	Non-RDQ	2.17	1.18	
My living situation allows me to focus on my studies.	RDQ	3.82	1.00	<.001**
Meine Lebenssituation erlaubt mir, mich auf mein Studium zu konzentrieren.	Non-RDQ	4.22	0.90	

Appendix E: Proportion of sources of study funding

2-sample test for equality of proportions with continuity correction, two sided: n = 359; RDQ: n = 158, Non-RDQ: n = 201. ** = <.01.

**Item**	**RDQ**	**Non-RDQ**	***p***
Federal student financial aid, dependent from parents' income<br>*Elternabhängiges BAföG*	8%	14%	0.085
Federal student financial aid, independent from parents' income<br>*Elternunabhängiges BAföG*	6%	3%	0.313
Scholarship (one or more)<br>*Stipendium (eines oder mehrere)*	8%	13%	0.211
Parents/relatives<br>Eltern/Angehörige	72%	85%	0.004**
My partner<br>*Meine Partnerin / mein Partner*	14%	2%	<.001**
Employment in a job with completed vocational training<br>*Erwerbstätigkeit in Beruf mit abgeschlossener Ausbildung*	53%	19%	<.001**
Part-time job/ student side job (e.g.,catering)<br>*Nebenjob / Studentenjob (z.B. Gastronomie)*	28%	49%	<.001**
Other income<br>*Sonstige Einkünfte*	15%	9%	0.184
